# Continuous succinic acid production by *Actinobacillus succinogenes* on xylose-enriched hydrolysate

**DOI:** 10.1186/s13068-015-0363-3

**Published:** 2015-11-14

**Authors:** Michael F. A. Bradfield, Ali Mohagheghi, Davinia Salvachúa, Holly Smith, Brenna A. Black, Nancy Dowe, Gregg T. Beckham, Willie Nicol

**Affiliations:** Department of Chemical Engineering, University of Pretoria, Lynnwood Road, Hatfield, Pretoria, 0002 South Africa; National Renewable Energy Laboratory, National Bioenergy Center, 15013 Denver West Parkway, Golden, CO 80401 USA

**Keywords:** Biorefinery, *Actinobacillus succinogenes*, Succinic acid, Continuous fermentation, Corn stover hydrolysate

## Abstract

**Background:**

Bio-manufacturing of high-value chemicals in parallel to renewable biofuels has the potential to dramatically improve the overall economic landscape of integrated lignocellulosic biorefineries. However, this will require the generation of carbohydrate streams from lignocellulose in a form suitable for efficient microbial conversion and downstream processing appropriate to the desired end use, making overall process development, along with selection of appropriate target molecules, crucial to the integrated biorefinery. Succinic acid (SA), a high-value target molecule, can be biologically produced from sugars and has the potential to serve as a platform chemical for various chemical and polymer applications. However, the feasibility of microbial SA production at industrially relevant productivities and yields from lignocellulosic biorefinery streams has not yet been reported.

**Results:**

*Actinobacillus succinogenes* 130Z was immobilised in a custom continuous fermentation setup to produce SA on the xylose-enriched fraction of a non-detoxified, xylose-rich corn stover hydrolysate stream produced from deacetylation and dilute acid pretreatment. Effective biofilm attachment, which serves as a natural cell retention strategy to increase cell densities, productivities and resistance to toxicity, was accomplished by means of a novel agitator fitting. A maximum SA titre, yield and productivity of 39.6 g L^−1^, 0.78 g g^−1^ and 1.77 g L^−1^ h^−1^ were achieved, respectively. Steady states were obtained at dilution rates of 0.02, 0.03, 0.04, and 0.05 h^−1^ and the stirred biofilm reactor was stable over prolonged periods of operation with a combined fermentation time of 1550 h. Furthermore, it was found that a gradual increase in the dilution rate was required to facilitate adaptation of the culture to the hydrolysate, suggesting a strong evolutionary response to the toxic compounds in the hydrolysate. Moreover, the two primary suspected fermentation inhibitors, furfural and HMF, were metabolised during fermentation with the concentration of each remaining at zero across all steady states.

**Conclusions:**

The results demonstrate that immobilised *A. succinogenes* has the potential for effective conversion of an industrially relevant, biomass-derived feed stream to succinic acid. Furthermore, due to the attractive yields, productivities and titres achieved in this study, the process has the potential to serve as a means for value-added chemical manufacturing in the integrated biorefinery.

## Background

Sustainable production of fuels and chemicals is becoming increasingly important due to a growing global demand for energy, uncertainty in the supply of petroleum resources, and environmental concerns associated with petrochemicals processing. To this end, the lignocellulosic biorefinery concept plays an important role as it has the potential to produce fuels, chemicals, and power sustainably from terrestrial biomass [1, 2]. Especially in the last decade, intensive research and development efforts have been conducted to realise industrial-scale lignocellulosic ethanol, and these facilities are currently becoming operational. Moving forward, it is expected that the economic viability of these biorefineries can be greatly improved by the production of chemicals alongside fuels (i.e. co-production), similar to current petroleum refineries where an array of products are produced at a single, integrated facility. Specifically, the production of fuels in a petroleum refinery enables economies of scale which lowers overall facility costs, while co-production of value-added chemicals can bolster the economics and increase profitability [3]. Manufacturing valuable chemicals will provide a similar advantage to lignocellulosic biorefineries by reducing the risks inherent in the economics of the overall process. As an alternative to petroleum-based routes, several value-added chemicals are currently being produced at scale as standalone processes using starch- or sugarcane-derived, sugar streams instead of lignocellulosic sugars. To realise industrial co-production of chemicals from biomass in a cost-effective manner will require detailed integration of unit operations from biomass deconstruction, sugar upgrading, separations, and product upgrading and finishing, warranting a co-design, integrated approach. Therefore, it still remains to be seen exactly how all the unit operations will be combined and what the optimal process synthesis schemes will be, but co-production of chemicals certainly has an important role to play in the success of biorefineries. Also, given the size disparity between fuel and chemical markets, multiple co-production strategies and careful product selection will be necessary at significant deployment of industrial-scale, integrated biorefineries.

Succinic acid (SA)—a four-carbon, aliphatic, dicarboxylic acid—has been identified as one of the top potential value-added chemicals from biomass due to its potential as a chemical precursor, and because it can be readily produced from biological transformation of biorefinery sugars since it is an intermediate in the tricarboxylic acid cycle [4–6]. The existing applications of SA lie in the food, pharmaceuticals, and chemicals industries, and due to its functional groups it can be catalytically converted to a variety of intermediates such maleic anhydride, 1,4-butanediol, tetrahydrofuran, γ-butyrolactone, and adipic acid [7]. Furthermore, SA has the potential to serve as a substrate for the production of bio-based polymers such as polybutylene succinate, among other polyesters [8], thereby expanding the SA market size and pull. Going forward, commercial success of fermentative SA production in an integrated biorefinery context will depend on the ability to integrate with upstream and downstream processing steps.

Current commercial bio-production of SA is based largely on pure sugar feed streams derived from starch-based raw materials such as corn and sorghum, resulting in a dependence on a feedstock that potentially competes with food resources—with the one exception being the possible use of glycerol as a carbon source. The commercial processes utilise engineered yeasts (*Saccharomyces cerevisiae*, *Candida krusei*) or modified bacteria (*Basfia succiniciproducens*, *Escherichia coli*) [9] as the microbial platforms. At the bench scale, various bacteria have been explored for SA production and competitive yields, titres and productivities on pure sugar substrates have been demonstrated with *Anaerobiospirillum succiniciproducens* [10, 11], *Actinobacillus succinogenes* [12–15], *Mannheimia succiniciproducens* [16–18] and engineered strains of *E. coli* [19–22]. Of particular interest to the current study, *A. Succinogenes* naturally produces succinate at considerable titres in mixed-acid fermentations [23, 24], and naturally and unavoidably forms biofilm which increases cell density in the fermenter thereby enhancing productivity [12, 14, 15, 25–28]. Moreover, *A. succinogenes* is a facultative anaerobe that grows optimally at high CO_2_ concentrations, and indeed, CO_2_ fixation is required for SA production. This, in combination with its ability to consume a broad range of biorefinery sugars (e.g. glucose, xylose, arabinose, galactose) [24] and its high acid tolerance [29], makes *A. succinogenes* a promising candidate for industrial succinate production on lignocellulosic feedstocks.

Given the ability of *A. succinogenes* to utilise hemicellulose-derived sugars such as xylose and arabinose, multiple bench-scale SA production studies have been conducted with this microbe using mixed sugar streams enriched in xylose, including from corn stover hydrolysate [30], straw hydrolysate [31], and sugar cane bagasse [32]. In an integrated biorefinery, obtaining a xylose-enriched stream can be accomplished via acid or hydrothermal pretreatment approaches, which fractionates biomass into a cellulose-rich stream to proceed to enzymatic hydrolysis and a xylose-rich hydrolysate primarily derived from hemicellulose breakdown. Even though these previous studies employ renewable feedstocks, the pretreatment methods lack process relevance as they typically employ batch, autoclave-type reactions with sulphuric acid. One particular pretreatment process, namely the combination of deacetylation, which is a mild alkaline wash, and continuous dilute acid pretreatment, is capable of producing high yields of monomeric xylose and other hemicellulose-derived sugars at the pilot scale today [33–35]. The resulting xylose-enriched stream can be separated from the remaining cellulose-enriched solids [36], and used as a distinct process stream for chemicals or fuels production within a biorefinery. Salvachúa et al. [37] employed such a pretreatment process, with and without the deacetylation step, on corn stover to produce a xylose-enriched hydrolysate stream from which competitive SA production by *A. succinogenes* was demonstrated in batch fermentations. In addition, mock hydrolysate streams were used to further explore the effects of potential microbial inhibitors. The release of microbial inhibitors such as furfural, hydroxymethylfurfural (HMF), acetic acid, and low molecular weight phenolic compounds is an additional major consideration of pretreatment processes, other than sugar yields, that is highly relevant to the co-design aspect for downstream fermentation [38]. A downstream fermentation process must necessarily tolerate the presence of these inhibitors or detoxification is required prior to fermentation. On the other hand, the integration benefits of the biorefinery model allow more effective utilisation of all process streams. For example, CO_2_ generated from biofuels production can be used to supplement the CO_2_ required in SA fermentations. Additionally, more cost-effective nitrogen sources necessary for microbial growth could be available within the biorefinery. Typically, *A. succinogenes* fermentations use a combination of yeast extract and corn steep liquor. Scale-up of yeast extract usage will be expensive, whereas corn steep liquor may be a suitable and more cost-effective replacement [39], especially since it is conceptually possible to produce this in a biorefinery or at least in conjunction with corn-based feedstock processing.

In view of the above context, it is therefore critical to explore fermentation processes that are suitable for inclusion in a biorefinery and that generate value-added chemicals through conversion of process-relevant, lignocellulosic feed streams. In previous work, we demonstrated batch production of succinic acid by *A. succinogenes* on several mock- and corn stover hydrolysates including from pilot-scale deacetylation and dilute acid pretreatment, and dilute acid pretreatment only [37]. Despite achieving competitive titres and yields without detoxifying the feed, the overall succinic acid production rates were limited. One option to enhance the productivity of a fermentation is to operate continuously with an immobilised cell reactor due to the distinctive productivity benefits that can be achieved through high cell concentrations [40]. Furthermore, immobilised cell reactors offer the potential advantages of long-term [41] and economical operation [40]. Regarding *A. succinogenes*, increased yields on clean sugar streams and enhanced SA productivities have been demonstrated with continuous operation of both biofilm systems [12–15, 27] and an external membrane cell recycle system [25]. In addition to the increased productivities offered by continuous operation, steady-state conditions allow for improved analysis of metabolite distributions and mass balance closure.

To this end, the present study explores continuous SA production by the conversion of a process-relevant, deacetylated, dilute acid pretreated corn stover hydrolysate stream using immobilised *A. succinogenes* 130Z as the biocatalyst. The xylose-enriched hydrolysate was separated from the cellulose-enriched solids after deacetylation with NaOH and dilute acid pretreatment with H_2_SO_4_ to provide a fractionated biorefinery stream rich in xylose, glucose, and other minor sugars. Fermentations were performed at various dilution rates in a stirred, biofilm reactor on a rich growth medium supplemented by continuous sparging of CO_2_. The bioreactor included a custom agitator fitting consisting of a porous polypropylene base and support arms, to facilitate and enhance biofilm attachment without compromising mixing. The hydrolysate stream was untreated prior to fermentation and so contained various putative fermentation inhibitors such as furfural, HMF, acetic acid and phenolic compounds.

## Results and discussion

### Reactor design

**Fig. 1:**
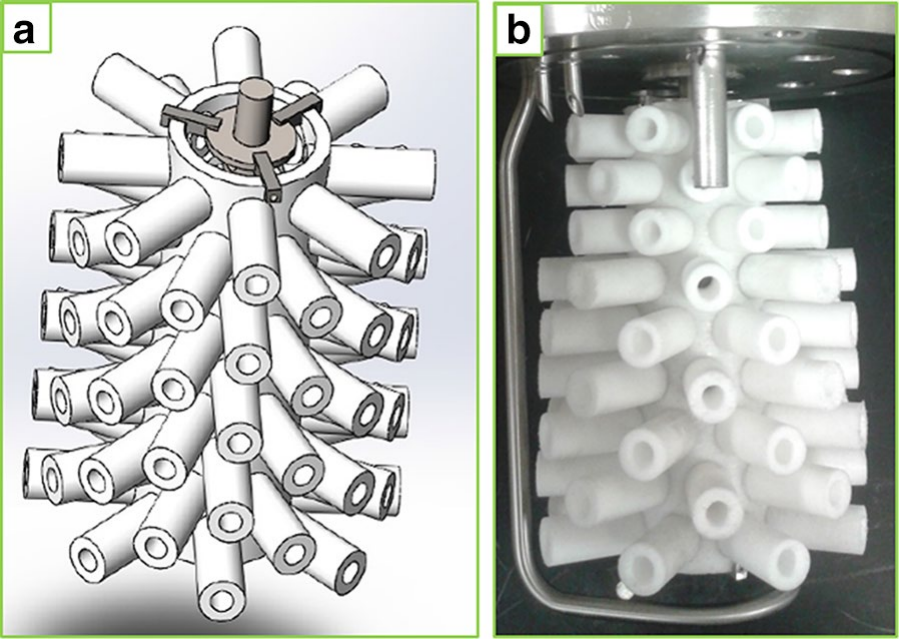
The agitator fitting used to increase cell density in the fermenter. **a** A 3D rendering of the fitting with polypropylene (PP) protruding arms used to increase biomass/cell density in the fermenter; **b** PP fitting attached to the agitator shaft

The bioreactor used for continuous fermentations consisted of a standard BioFlo 3000 fermenter (New Brunswick Scientific, USA) outfitted with a novel agitator fitting to support biofilm attachment and development (Fig. 1). The fitting comprised a central porous polypropylene (PP) tube perforated with a multitude of holes into which porous PP or silicone arms were affixed. The central tube was attached to the agitator shaft by means of stainless steel brackets allowing for easy detachment. The basis for the design was to provide additional surface area for biofilm attachment and support whilst achieving sufficient mixing and homogeneity of the fermentation broth through stirring. For example, the surface area-to-volume ratio of the reactor increased from 0.34 cm^2^ cm^−3^ (excluding the agitator fitting) to 1.36 and 1.31 cm^2^ cm^−3^ when using the silicone and PP fittings (excluding porosity), respectively. In addition, stirring would provide liquid circulation through the arms via the central tube thereby enhancing liquid flow through the internal regions of the fitting. The design was tested in the initial xylose fermentation run, using silicone protruding arms, and due to positive results, it was also employed in the hydrolysate fermentations where porous PP arms were used to further increase surface area since good attachment to the central PP tube was demonstrated in the xylose run.

### Continuous fermentation using xylose

An initial continuous fermentation was performed using a pure xylose feed stream (inhibitor free) to establish a baseline against which the hydrolysate fermentation runs could be compared, as xylose is the major sugar in dilute acid pretreated hydrolysates [35, 42–44]. A xylose feed concentration of 60 g L^−1^ was chosen since this was the estimated concentration of xylose in the mock and hydrolysate runs (80 g L^−1^ total sugars) of our comparative batch study [37]. A second objective of the initial fermentation was to ensure that the novel agitator fitting was capable of facilitating biofilm attachment and support, preferably for extended periods of operation.

Steady states were achieved at dilution rates (D) of 0.05 and 0.10 h^−1^ (Table 1) with succinic acid as the major product, and acetic acid (AA) and formic acid (FA) as by-products. The productivity benefits of operating continuously with a biofilm reactor are highlighted when comparing the SA productivity attained in this study (1.5–2.6 g L^−1^ h^−1^) to that of our previous xylose batch study (60 g L^−1^ feed) of 0.94 g L^−1^ h^−1^ [37] and to a similar study by Liu et al. [45] of 0.54 g L^−1^ h^−1^. Furthermore, the productivity compares similarly to the other previous continuous fermentations of xylose by *A. succinogenes* [46] where productivities of 1.5–3.4 g L^−1^ h^−1^ were attained at Ds between 0.05 and 0.3 h^−1^. Productivity is calculated using the overall reactor volume (1.3 L) and the volumes of the agitator and fitting form part of this volume.

**Table 1:** Summary of the biofilm reactor performance on a clean xylose stream

Dilution rate (h^−1^)	Productivity (g L^−1^ h^−1^)	SA (g L^−1^)^a^	Yield on xylose (g g^−1^)	SA/AA (g g^−1^)^b^	FA/AA (g g^−1^)^c^	Sugar conversion (%)	Effective xylose feed (g L^−1^)
0.05	1.54	32.5	0.72	4.2	0.36	80	57.0
0.10	2.64	26.4	0.77	4.2	0.37	60	56.7

^a^Succinic acid

^b^Acetic acid

^c^Formic acid

**Fig. 2:**
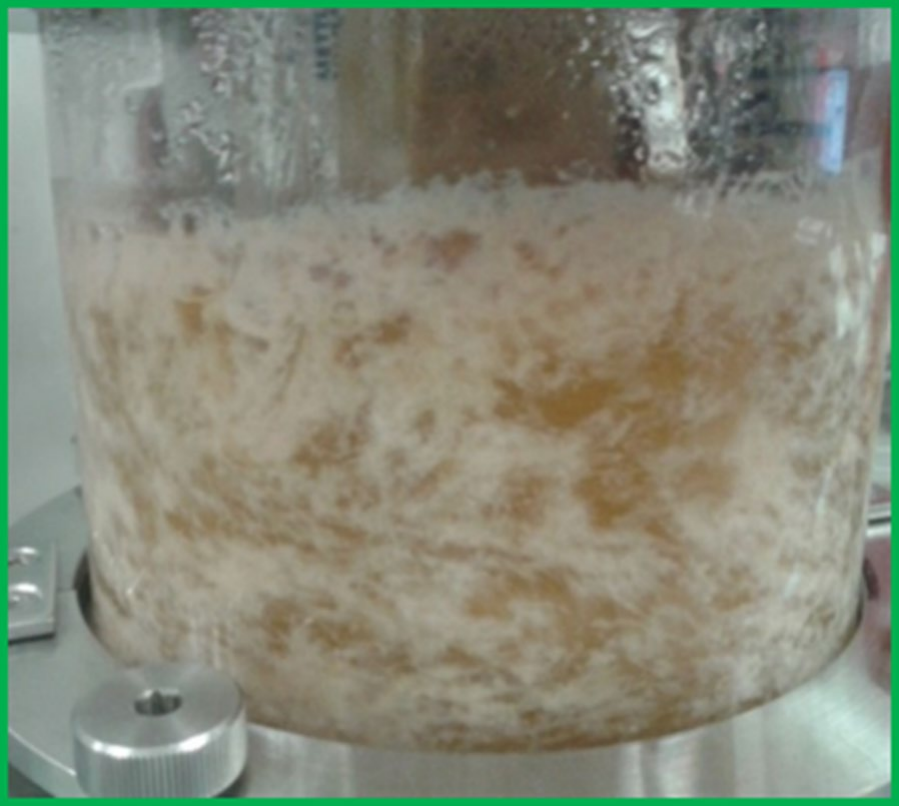
Biofilm of *A. succinogenes* grown on xylose. Biofilm attachment occurred on the walls of the fermenter and the agitator which is not visible due to the density of the biofilm and the opacity of the broth

The maximum SA titre in this study (32.5 g L^−1^) is lower than that of our previous batch study (38.4 g L^−1^), whereas the maximum yield is greater (0.77 vs 0.70 g g^−1^) and both values compare remarkably well with that of Liu et al. [45] (32.6 g L^−1^ and 0.77 g g^−1^), thereby further motivating continuous operation. In addition, it was found that the agitator fitting effectively facilitated biofilm attachment and support during continuous operation. Biofilm attached to all surfaces of the silicone protruding arms and the inner tube of the fitting, and also to the glass walls and internals of the fermenter (Fig. 2). Prior to inoculation, the pH response to base addition was found to be rapid, which suggests that effective mixing was achieved by the agitator. Therefore, the agitator fitting provided adequate support for biofilm and did not compromise mixing of the broth. In addition, minimal loss of cells was observed in the reactor effluent, further indicating the effectiveness of the fitting.

### Continuous fermentation using hydrolysate (DDAP-H): effect of dilution rate

Following the promising results of the pure xylose fermentations, continuous fermentations of xylose-enriched, deacetylated, dilute acid pretreated corn stover hydrolysate (DDAP-H) were performed. The DDAP-H was prepared by a two-stage pretreatment of corn stover comprising a mild alkaline wash with NaOH followed by dilute acid pretreatment (DAP) with H_2_SO_4_ (see “Methods”). Deacetylation is effective at removing a significant amount of acetic acid from the hydrolysate [33], which is beneficial since acetic acid is known to be inhibitory to the growth of *A. succinogenes* [29, 47]. Deacetylation also partially removes lignin from the cell wall, which potentially could reduce inhibition due to low molecular weight phenolics. The DDAP-H consisted mainly of C_5_ and C_6_ carbohydrates at a total concentration of 104.8 g L^−1^ along with fermentation inhibitors such as furfural and HMF (originating from sugar degradation during DAP [38]), and acetic acid. Although the expected xylose concentration in the hydrolysate streams of the comparative batch study was 60 g L^−1^ (with a corresponding total sugars concentration 80 g L^−1^), fluctuations in the total and relative sugar concentrations between hydrolysate batches resulted in an actual xylose feed concentration of between 52 and 58 g L^−1^. Therefore, in the present study, the pressed hydrolysate was diluted (Table 2) to achieve an operational xylose concentration within this range for direct comparison with the DDAP-H results of the comparative study [37].

**Table 2:** Composition of the diluted DDAP-H in the fermentation medium as fed to the fermenter

Compound	Feed (g L^−1^)
Glucose	8.3
Xylose	52.6
Galactose	3.7
Arabinose	5.3
Acetic acid	0.9
Furfural	0.62
HMF	0.11

Undiluted DDAP-H constituted approximately 65 % v/v of the fermentation medium

**Fig. 3:**
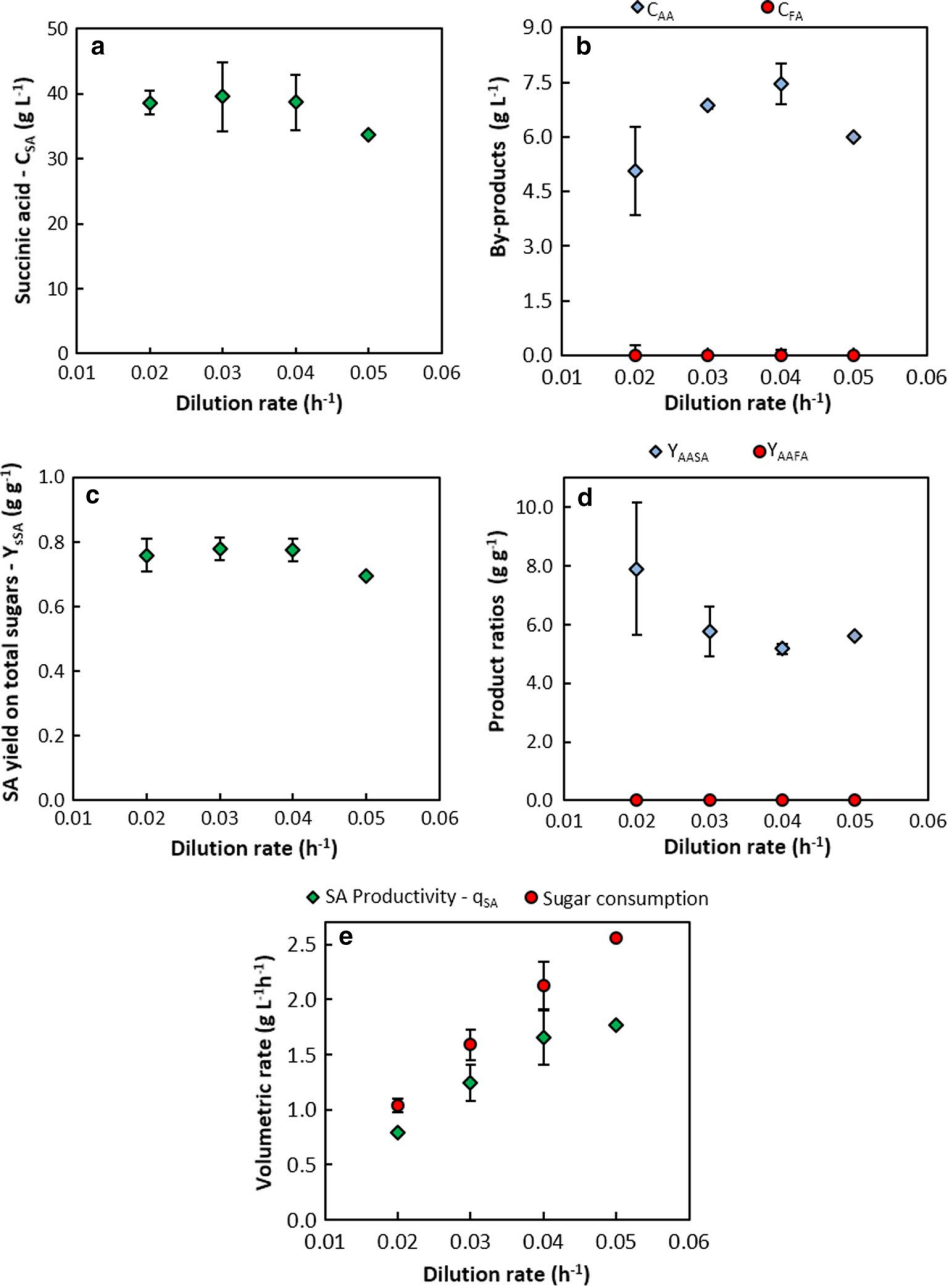
Fermentation performance on DDAP-H as a function of dilution rate. Concentrations of **a** succinic acid, and **b** the major by-products acetic acid and formic acid; **c** the yield of succinic acid on total sugars consumed; **d** metabolite ratios indicating selectivity to succinic acid (*Y*_AASA_) and the route of pyruvate consumption (*Y*_AAFA_); **e** volumetric rate of carbohydrate consumption and succinic acid productivity. *Error bars* represent standard deviation and are hidden by the *markers* in cases where the deviation is negligible. No repeats were performed at *D* = 0.05 h^−1^

The DDAP-H fermentations were performed in duplicate at dilution rates of 0.02, 0.03 and 0.04 h^−1^. Steady states were achieved at all three dilution rates with a single steady state achieved at a dilution rate of 0.05 h^−1^ to gain insight into the response of the system at higher dilution rates. The dynamic behaviour of the system, discussed further below, was instructive in selecting optimal dilution rates, in developing a start-up procedure for stable continuous operation and in assessing steady-state stability. It was found that the succinic acid concentration (C_SA_) remained fairly constant at between 38.6 and 39.6 g L^−1^ on average across all three duplicated dilution rates (Fig. 3a), and decreased to 33.7 g L^−1^ at *D* = 0.05 h^−1^. However, *C*_SA_ was shown to increase with decreasing dilution rate in previous studies with *A. succinogenes* [12–14], although these studies did not include all the low Ds used in the current study. Despite the constant C_SA_ values, the concentrations of the major by-products, acetic acid (*C*_AA_) and formic acid (*C*_FA_), showed similar trends to previous continuous fermentations where *C*_AA_ decreased with decreasing *D*, and *C*_FA_ remained near, or equal, to zero (Fig. 3b), with the only exception being the concentration at *D* = 0.05 h^−1^ where *C*_AA_ decreased along with *C*_SA_.

Similar to the trend in *C*_SA_, the yield of SA on carbohydrates (*Y*_sSA_) remained fairly constant (0.76–0.78 g g^−1^) across the lower three dilution rates and decreased to 0.69 g g^−1^ at *D* = 0.05 h^−1^ (Fig. 3c). Furthermore, the yields remained below the overall theoretical maximum of 1.12 g g^−1^ [21] (on a glucose basis, but this holds for all carbohydrates in this study due to the same degree of reduction seen in the identical C:H:O ratios), and within the limits defined by the accepted metabolic pathways of 0.66–0.87 g g^−1^ [15]. Despite this, the SA/AA ratios (*Y*_AASA_) exceeded those dictated by the same pathways (1.97 and 3.93 g g^−1^) and ranged between 5.2 and 7.9 g g^−1^ whereas the FA/AA ratio (*Y*_AAFA_) remained constant at zero due to the absence of FA (Fig. 3d). Since *C*_SA_ remained constant at a constant *Y*_sSA_ with a corresponding decrease in *Y*_AASA_ at increasing Ds, it implies that carbon was increasingly channelled to AA but not away from SA. However, in the case of a constant yield when all metabolites are accounted for, it is expected that as *C*_AA_ increased, *C*_SA_ should have decreased.

The SA productivity (*q*_SA_) was competitive and ranged between 0.78 and 1.65 g L^−1^ h^−1^ for the lower three dilution rates (Fig. 3e). In addition, *q*_SA_ increased linearly up to a *D* of 0.04 h^−1^ due to *C*_SA_ remaining fairly constant, but flattened out somewhat at *D* = 0.05 h^−1^ due to a correspondingly lower *C*_SA_. The total sugars consumption rate increased linearly across all dilution rates including 0.05 h^−1^. The highest *q*_SA_ of 1.77 g L^−1^ h^−1^ was achieved at *D* = 0.05 h^−1^ with a corresponding total sugars consumption rate of 2.56 g L^−1^ h^−1^. The decrease in *Y*_sSA_ at *D* = 0.05 h^−1^ is also reflected by the increased difference between the rate of sugars consumption and the rate of SA production when compared to the lower Ds. The non-linear increase in *q*_SA_ in moving from a D of 0.04–0.05 h^−1^ with a corresponding linear increase in the rate of sugar consumption, together with a poorer yield at 0.05 h^−1^, is suggestive of a shift in the metabolic flux distribution.

**Fig. 4:**
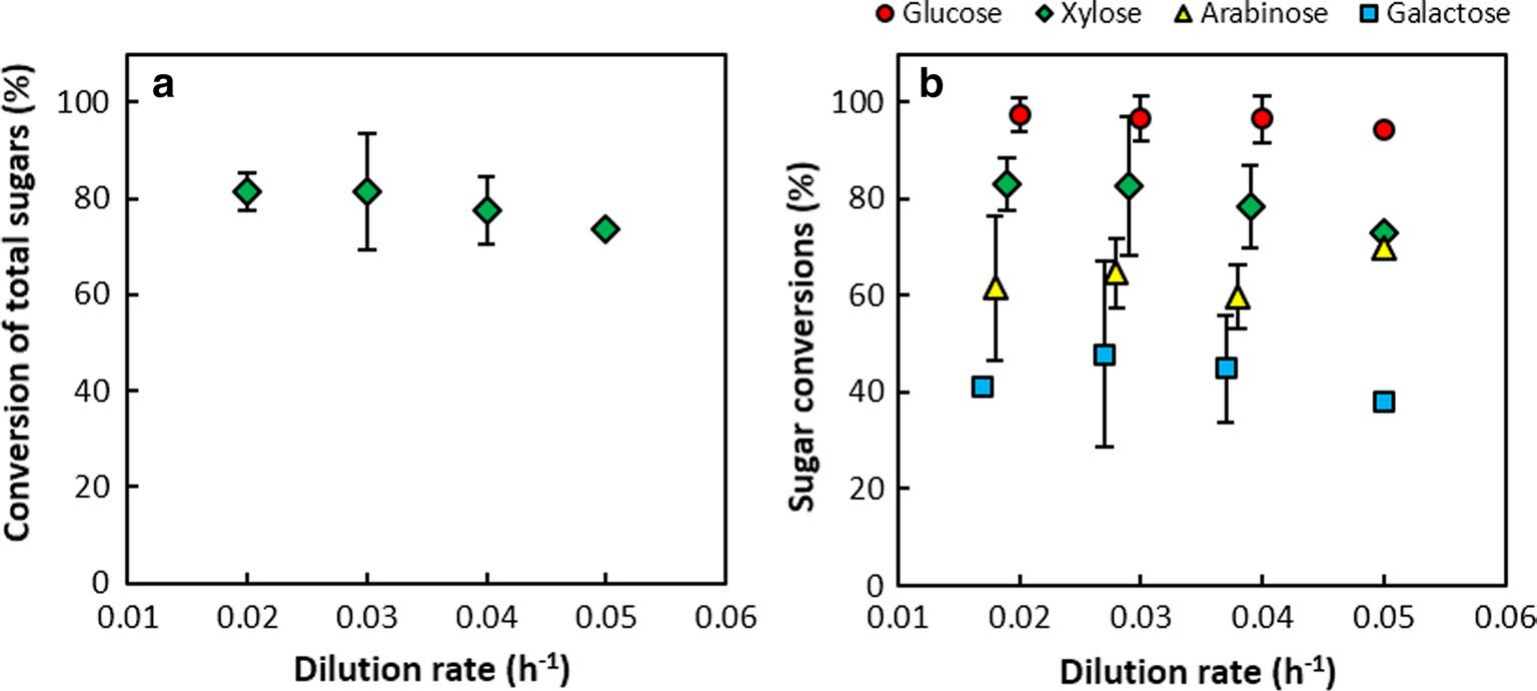
Conversion of carbohydrates in the DDAP-H fermentations as a function of dilution rate. **a** The conversion of total sugars, and **b** the individual conversion of each sugar. *Error bars* represent standard deviation and are hidden by the *markers* in cases where the deviation is negligible. No repeats were performed at *D* = 0.05 h^−1^. Large deviations in arabinose and galactose data are due to reduced HPLC system sensitivity at low sugar concentrations

Despite the linear increase in sugar consumption rate with dilution rate, the conversion of total sugars decreased gradually with increasing dilution rate from 81.3 % at *D* = 0.02 h^−1^ to 73.7 % at *D* = 0.05 h^−1^ (Fig. 4a). The order of preference in sugar utilisation by *A. succinogenes*, as reflected by the conversion of each sugar (Fig. 4b), was glucose (94.3–97.5 %), xylose (73.0–83.2 %), arabinose (61.5–70.0 %) and galactose (37.9–47.9 %). However, all sugars were consumed simultaneously suggesting the absence of carbon catabolite repression, in agreement with the results of our previous batch study on DDAP-H [37] where the sugars were consumed simultaneously but at different rates with the same utilisation preferences as seen in the present study. Interestingly, the conversion of xylose at 0.05 h^−1^ in the DDAP-H fermentation (73.0 %) was somewhat lower than that of the baseline xylose fermentation at the same *D* (79.6 %) with a similar xylose feed concentration—likely due to the preference for glucose.

### Continuous fermentation using hydrolysate (DDAP-H): mass and redox balance analyses

**Fig. 5:**
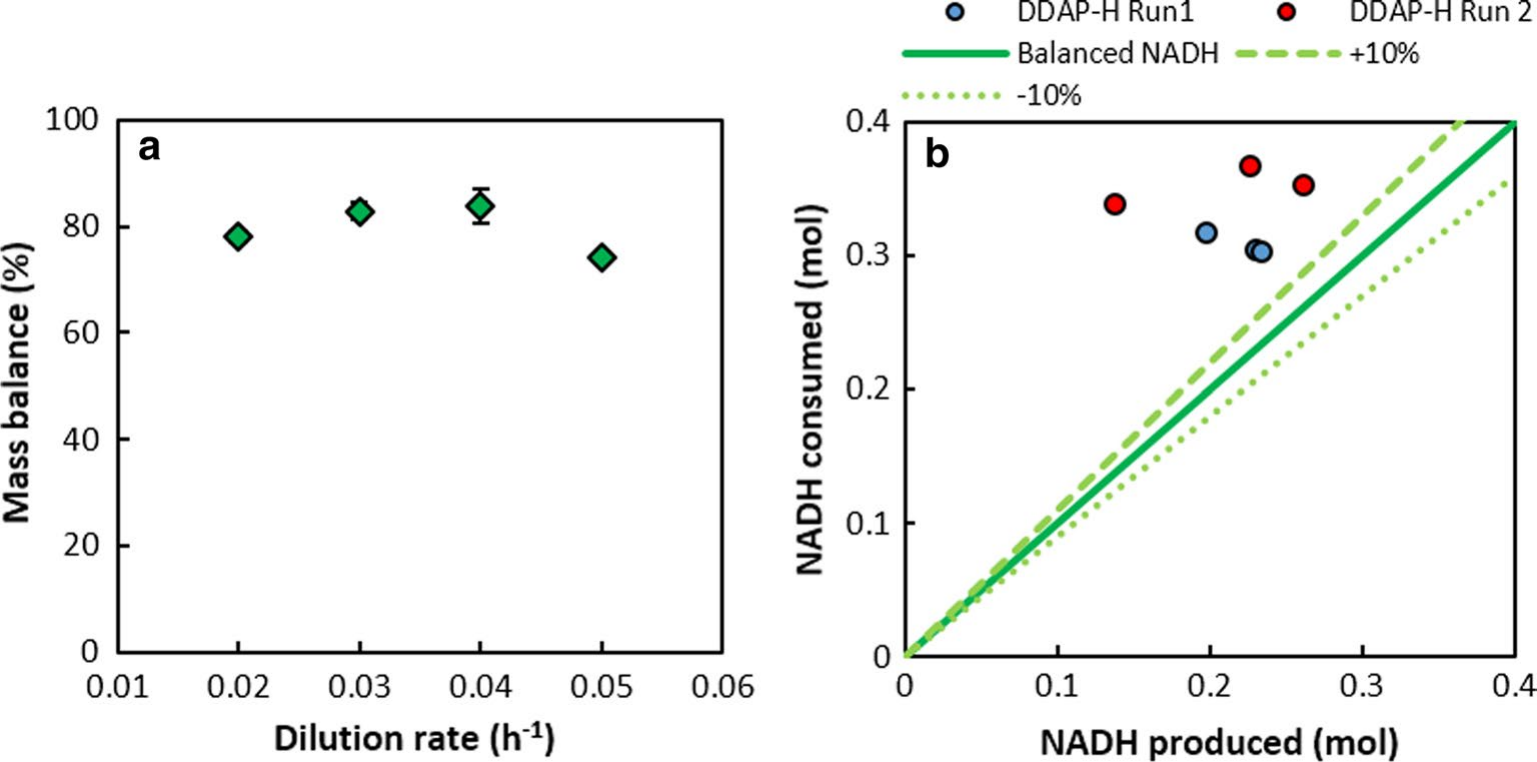
Mass and redox balance analyses of the DDAP-H fermentations. **a** Mass balances at each dilution rate and, **b** a parity plot of the NADH produced as a function of the NADH consumed for each steady state from DDAP-H runs 1 and 2. *Error bars* represent standard deviation and are hidden by the *markers* in cases where the deviation is negligible. No repeats were performed at *D* = 0.05 h^−1^

To explore the consistency of the data, mass balances (see *Methods*) were performed on the data averages. Mass balance closures were between 74.0 and 83.9 % (Fig. 5a) suggesting that mass was unaccounted for in the form of either missing metabolites or as biomass since dry cell weights were not included. Biomass, as dry cell weight measurements, was excluded from the mass balance calculation due to the presence of biofilm, which could not be quantified in real time without complete termination of the fermentation, and due to shedding and sloughing of biofilm, which would influence the suspended cell readings. However, the growth rate of *A. succinogenes* has been shown to decrease significantly with increasing *C*_SA_ and tends towards zero at a *C*_SA_ above 15 g L^−1^ [14]. Furthermore, in a previous study on *A. succinogenes*, it was shown that over steady-state periods of 24 h (*D* = 0.05 h^−1^) with an established biofilm, dry cell weights were 0.19 g L^−1^ on average with associated glucose consumptions ranging between 40 and 44 g L^−1^ at *C*_SA_ values between 33.8 and 34.5 g L^−1^ [15]. Under these conditions, the system was in a non-growth mode with associated maintenance-based succinic acid production. This implies that at most 0.45 ± 0.03 % of the total glucose consumed was channelled to biomass, thus highlighting the trivial contribution of biomass to the overall mass balance under appreciable SA titres. Based on these two observations, and since *C*_SA_ values at steady state were between 33.0 and 39.5 g L^−1^ throughout this study, it was assumed that carbon flux to biomass was negligible. Therefore, the unaccounted mass is likely due to undetected metabolites, which may have been produced through alternative metabolic pathways. *A. succinogenes* lacks a complete TCA cycle due to the absence of the genes encoding isocitrate dehydrogenase and citrate synthase in the oxidative branch of the cycle, and also lacks a glyoxylate shunt [48]. Therefore, the only route of SA synthesis is via the reductive branch of the TCA cycle. Besides succinic acid, the main reported end products during *A. succinogenes* fermentations are acetic acid, formic acid and ethanol [49]. In addition, intermediates in the TCA cycle (i.e. fumarate, malate, oxaloacetate and citrate) have not been observed in previous studies of *A. succinogenes* or in our laboratory, despite a report of citrate lyase activity in *A. succinogenes* cell extracts [49]. α-Ketoglutarate synthesis has also been ruled out [50].

The incomplete mass balances do not detract from the utility of the fermentation results and are in themselves a useful result, yet it is useful to further explore this discrepancy and to this end, redox balances were performed on the results. For simplicity, reduced cofactors are lumped as NADH since the same amount of carbon is used to provide reduced cofactors and the yield on substrate will be the same. Production and consumption of NADH can be calculated from the overall metabolic pathways in converting carbohydrates to metabolites as outlined in Villadsen et al. [51]. Overall pathways are essentially a summation of all the intermediate metabolic reactions involved in converting substrate into the specified end product. A simplified metabolic network based on Bradfield and Nicol [15] was used to determine the overall pathways and since the degree of reduction of each carbohydrate is the same, the redox implications will be equivalent for each. A complete depiction of the central metabolism and the carbohydrate uptake pathways of *A. succinogenes* is provided by McKinlay et al. [48].

Equations 1 and 2 (carbon mole basis) give the overall oxidative pathways in which NADH is produced, via either the pyruvate dehydrogenase (Eq. 1) or the pyruvate formate lyase (Eq. 2) route. However, since no formic acid was observed during the DDAP-H fermentations, only Eq. 1 featured in the NADH calculation. Equation 3 gives the overall reductive pathway in which there is a net consumption of NADH in producing SA. Using metabolite measurements, the corresponding NADH for each pathway can be calculated and the total should sum to zero in a redox balanced system. However, in this study, the total NADH consumed far exceeded the NADH produced (Fig. 5b), which dovetails with the high *Y*_AASA_ ratios (Fig. 3d). The extent to which the consumed NADH exceeded the produced NADH clearly reflects that NADH generation from production of the measured metabolites was insufficient. In essence, therefore, the system displayed an overall mass imbalance together with an NADH imbalance on the measured mass.

1 $${\text{CH}}_{2} {\text{O}}\left( {\text{carbohydrates}} \right) + \frac{1}{3}{\text{H}}_{2} {\text{O}} \to \frac{2}{3}{\text{CH}}_{2} {\text{O}}\left( {\text{AA}} \right) + \frac{2}{3}{\text{NADH}} + \frac{1}{3}{\text{CO}}_{2}$$

2 $${\text{CH}}_{2} {\text{O}}\left( {\text{carbohydrates}} \right) + \frac{1}{3}{\text{H}}_{2} {\text{O}} \to \frac{2}{3}{\text{CH}}_{2} {\text{O}}\left( {\text{AA}} \right) + \frac{1}{3}{\text{CH}}_{2} {\text{O}}_{2} \left( {\text{FA}} \right) + \frac{1}{3}{\text{NADH}}$$

3 $${\text{CH}}_{2} {\text{O}}\left( {\text{carbohydrates}} \right) + \frac{1}{3}{\text{NADH}} + \frac{1}{3}{\text{CO}}_{2} \to \frac{4}{3}{\text{CH}}_{{\frac{3}{2}}} {\text{O}}\left( {\text{SA}} \right) + \frac{1}{3}{\text{H}}_{2} {\text{O}}$$

The mass deficit could be associated with NADH production where NADH is produced together with the suspected missing metabolite(s), thereby accounting for both the excess NADH and the missing mass. It is also plausible that additional NADH was produced via the oxidative pentose phosphate pathway (OPPP) as recently proposed by Bradfield and Nicol [15]. The OPPP produces NADPH and CO_2_, and since transhydrogenase activity has been detected in *A. succinogenes* [52], NADPH can be oxidised to NADP^+^ with concomitant reduction of NAD^+^ to NADH. However, OPPP flux would only satisfy the redox balance while leaving the overall mass balance unsatisfied. On the other hand, if the OPPP produced NADH beyond that needed to close the NADH balance, and the additional NADH produced was oxidised by an external agent (e.g. yeast extract in the feed serving as an electron acceptor as mentioned in [53]), a concomitant loss of CO_2_ would occur. In this scenario, the undetected metabolite would be CO_2_.

### Effect of inhibitors on the fermentation performance

The concentrations of the primary suspected fermentation inhibitors, furfural and HMF, were found to decrease to, and remain at zero, during all fermentations. This finding is in agreement with our previous batch study where furfural was converted to furfuryl alcohol and consequently decreased to zero, together with HMF, during the course of the fermentation [37]. As suggested in a previous study [54], furfural conversion to furfuryl alcohol likely occurs by means of an aldehyde reductase since the aldehyde is reduced to its alcohol form. Also, the genome of *A. succinogenes* encodes an aldo/keto reductase (KEGG: Asuc_0311), which may be responsible for the reduction of furfural. Despite HMF and furfural remaining at zero, it was necessary to increase the dilution rate gradually to enable the culture to better tolerate the hydrolysate (see “Dynamic behaviour of the hydrolysate (DDAP-H) fermentation: start-up and stability”) suggesting either the presence of other inhibitors in the feed, or that *A. succinogenes* metabolises these compounds at a regulated rate that increases with increasing dilution rate after adaptation. Similarly, in our comparative batch study [37], the mock hydrolysates that contained furfural and HMF distinctly outperformed the actual hydrolysates suggesting that there are inhibitors present in the hydrolysate besides HMF and furfural. Phenolic compounds resulting from hydrolysis pretreatment processes are also known to inhibit microbial growth [38]. As such, the concentrations of selected phenolic compounds in the feed were compared to those of the fermentation broth across all steady states in the second DDAP-H fermentation (Table 3). Interestingly, an increase in the concentrations of the phenolics was observed between the feed and the fermentation broth, with the exception of 4-hydroxybenzaldehyde. Also, the increase in phenolics occurred at all dilution rates and to the same extent. An increase in phenolics could be a result of breakdown of lignin oligomers or aromatic-carbohydrate linkages, either through microbial action or through abiotic degradation. Related to microbial action, the genome of *A. succinogenes* includes a feruloyl esterase enzyme (KEGG: Asuc_0433) which is able to catalyse the breakdown of the complex feruloyl-polysaccharide thereby releasing ferulate (Expasy: EC 3.1.1.73). Because the enzyme contains a signal peptide (predicted by SignalP 4.1 [55]), it may be performing extracellular hydrolysis reactions, which could be the mechanism behind the increase in ferulic acid. In spite of this potential mechanism, it remains to be seen whether overall fermentation performance can be enhanced by detoxification of the hydrolysate prior to fermentation without considerably impacting the economics of the process. We will examine hydrolysate detoxification for succinic acid production in a future study.

**Table 3:** Concentrations of phenolic compounds present in the feed and outlet during the second DDAP-H fermentation

Dilution rate (h^−1^)	(mg L^−1^)					
	4-Hydroxy benzaldehyde	Caffeic acid	Syringic acid	p-coumaric acid	Ferulic acid	Total phenolics
Feed	1.779	ND	ND	22.71	5.20	29.68
0.02	0.105	0.895	0.269	51.84	15.15	68.26
0.03	0.104	0.886	0.261	51.40	15.20	67.85
0.04	0.107	0.881	0.264	51.05	14.85	67.15
0.05	0.111	0.887	0.272	51.93	15.14	68.34

*ND* not detected

### Comparison to other relevant studies on succinic acid production

The results achieved in this study compare well with previous SA production studies using *A. succinogenes* and a biomass feedstock. Particularly, the highest productivity achieved in this study (1.77 g L^−1^ h^−1^) exceeds previous batch studies that utilised a lignocellulosic feedstock, a starch-derived feedstock, or a detoxified feed stream but was lower than the previous continuous xylose study (3.41 g L^−1^ h^−1^; at *D* = 0.3 h^−1^) (Table 4). As with the baseline xylose fermentation, the high productivities attained in the DDAP-H fermentations highlight the productivity benefits of operating continuously with immobilised cells. However, the maximum titre attained (39.6 g L^−1^) was lower than previous studies which underscores a potential downside to operating continuously at appreciable productivities. The maximum yield on total sugars attained in the DDAP-H fermentations of 0.78 g g^−1^ compares well with the xylose baseline fermentation (0.77 g g^−1^) and with previous studies, especially since the hydrolysate contained known fermentation inhibitors, and comprised a mix of sugars that are metabolised to varying degrees by *A. succinogenes* (Fig. 4b). Furthermore, the maximum yield in this study exceeded that of our previous comparative batch study [37]. Interestingly, the steady state, achieved at *D* = 0.05 h^−1^ in the second DDAP-H fermentation, outperformed that of the baseline xylose fermentation in terms of productivity (1.77 vs. 1.54 g L^−1^ h^−1^) and titre (33.6 vs. 32.5 g L^−1^), but not in terms of yield (0.69 vs. 0.72 g g^−1^). The greater productivity is most likely due to a higher cell density in the DDAP-H fermentation where the porosity of the PP fitting is expected to provide a greater surface area for cell attachment than the silicone arms used in the xylose fermentation. Also, the comparable performance could suggest that the lower initial, and gradual increase in, the dilution rates of the second DDAP-H fermentation (discussed below) together with increased exposure times of the cells to DDAP-H through cell retention led to adaptation of the culture to the extent where it was able to perform similarly to one operating on a clean, xylose feed—since the comparison is only valid for one dilution rate (0.05 h^−1^) which occurred late in the DDAP-H run. With this in mind, prior detoxification of the DDAP-H could lead to performance similar to that of the xylose fermentation without the need for extended start-up phases. Essentially, the results achieved in this study are of substantial value to the development of a SA production processes that could be incorporated into a lignocellulosic biorefinery. However, all studies on lignocellulosic feedstock still fall well short of the performance obtained with pure glucose fermentations (Table 4). In addition to *A. succinogenes*, the ability of other microorganisms to produce SA from lignocellulosic biomass has been reviewed [56].

**Table 4:** Summary of the most relevant succinic acid production studies using *A. succinogenes* and a potentially scalable, renewable feedstock with pure sugar studies given for comparison

Substrate (feedstock)	Pretreatment method	Mode	*q*_SA_ (g L^−1^ h^−1^)^a^	*C*_SA_ (g L^−1^)^b^	*Y*_sSA_ (g g^−1^)^c^	References
Corn stover (xylose, glucose, arabinose, galactose)	Pilot scale: deacetylation followed by dilute sulphuric acid hydrolysis	Continuous	1.77	39.6	0.78	This study
Xylose	–	Continuous	2.64	32.5	0.77	This study
Xylose	–	Continuous	3.41	29.4	0.68	[46]
Xylose (60 g L^−1^)	–	Batch	0.94	38.4	0.70	[37]
Corn stover	Pilot scale: deacetylation followed by dilute sulphuric acid hydrolysis (DDAP-H)		0.30	42.8	0.74	
	Pilot scale: dilute sulphuric acid hydrolysis (DAP-H)		0.12	17.0	0.52	
Mock DDAP-H (xylose, glucose, arabinose, galactose, inhibitors, low acetate)	–		0.61	43.8	0.68	
Mock DAP-H (xylose, glucose, arabinose, galactose, inhibitors, high acetate)	–		0.61	43.7	0.72	
Sugarcane bagasse (xylose)	Dilute sulphuric acid hydrolysis	Batch	1.01	22.5	0.43	[57]
Wheat (glucose)	Enzymatic hydrolysis	Batch	1.01	35.6	0.82	[58]
			1.57	64.2	0.81	[59]
			0.91	62.1	1.02	[60]
Corn fibre (glucose, xylose, arabinose)	Dilute sulphuric acid hydrolysis by autoclaving	Batch	0.98	35.4	0.73	[61]
			0.63	47.0	0.68	[62]
Corn stover (glucose, xylose, arabinose)	Dilute sulphuric acid hydrolysis followed by cellulose treatment	Batch	1.08	56.4	0.73	[30]
Sugarcane molasses (sucrose, glucose, fructose)	Sulphuric acid treatment of crude molasses	Batch	1.30	60.7	0.81	[63]
		Fed-batch	1.40	64.7	0.86	
		Batch	0.97	57.9	0.69	[64]
		Fed-batch	1.07	64.3	0.76	
Glucose^d^	–	Continuous	10.8	32.5	0.90	[12]
	–	Fed-batch	2.77	98.7	0.89	[65]

The highest SA productivity, concentration and yield for each study are shown

^a^Succinic acid productivity

^b^Succinic acid concentration

^c^Yield of succinic acid on carbohydrate substrate(s)

^d^Comparative baseline study

### Dynamic behaviour of the hydrolysate (DDAP-H) fermentations: start-up and stability

The initial operational strategy for the DDAP-H fermentations was to operate at a low dilution rate similar to the lowest rate used in the xylose baseline fermentation (0.05 h^−1^) to allow the culture to adapt to the hydrolysate. Given stability of the system, the dilution rate would then be increased to accelerate biofilm formation. Once substantial and stable biofilm had been established, the dilution rate would be systematically changed to assess the performance of the system under steady state conditions across a range of dilution rates. The system was considered to be at steady state once the time-averaged NaOH flow rate remained within 5 % of the average for at least 24 h and minimal fluctuations (<3 %) in residual sugar and metabolite concentrations were observed after at least two samples over the same period.

**Fig. 6:**
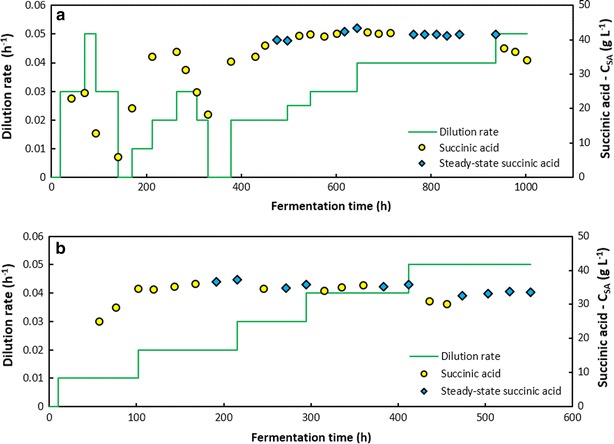
Dynamic behaviour of the DDAP-H fermentations. Time profiles of the dilution rate and succinic acid concentration for: **a** the first DDAP-H fermentation where too rapid an increase in the dilution rate led to washout, and **b** the second DDAP-H fermentation where the dilution rate was more gradually increased to facilitate adaptation of the culture to the hydrolysate

In the first continuous DDAP-H fermentation (Fig. 6a), once the initial start-up batch was nearing completion, the system was switched to continuous mode at a dilution rate of 0.025 h^−1^ (half of the lowest rate used in the xylose fermentation). The response was positive and a *C*_SA_ of 20 g L^−1^ was achieved. Given this, the dilution rate was increased to 0.05 h^−1^ before the system was able to reach steady state. However, the productivity of the system decreased significantly and ultimately approached zero. To restore the system, the reactor was switched to batch mode allowing the cells a period of revival. Batch operation showed an increase in *C*_SA_ after which the dilution rate was changed to 0.01 h^−1^ followed by an increase to 0.02 h^−1^ due to a promising increase in *C*_SA_. The dilution rate was then increased to 0.03 h^−1^ which resulted in a progressive decrease in *C*_SA_. Once again the system was switched to batch mode for recovery.

**Fig. 7:**
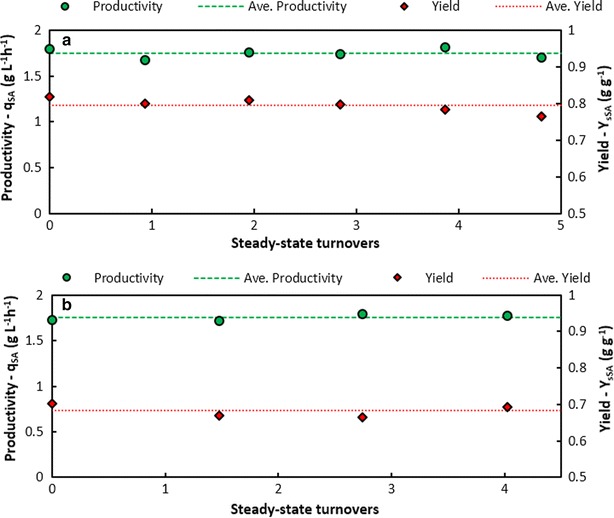
Steady-state stability of the DDAP-H fermentations. Time profiles of the succinic acid productivity and yield at steady state for: **a** the first DDAP-H fermentation at *D* = 0.04 h^‐1^ for almost five volume turnovers (120 h), where the slight decrease in yield and productivity towards the end of the time frame are due to biofilm shedding caused by minor pH control issues, and **b** the second DDAP-H fermentation at *D* = 0.05 h^−1^ for just over four volume turnovers (81 h). The points of each graph correspond to the consecutive steady-state points in Fig. 6

The increase in *D* from 0.01 to 0.03 h^−1^ may have been too rapid for the culture to adapt, thereby causing washout or cell death. Therefore, once the system had recovered, the dilution rate was switched to 0.02 h^−1^ and held for 120 h after which the first steady state was achieved at a *C*_SA_ of 39.6 g L^−1^. Subsequently, D was increased to 0.025 h^−1^ and then to 0.03 h^−1^. As evidenced by Fig. 6a, the more gradual increase in *D* resulted in an increase in *C*_SA_ up to a point where it plateaued over time and steady states were obtained at Ds of 0.03 and 0.04 h^−1^. After the switch in D from 0.03 to 0.04 h^−1^, the system exhibited steady behaviour after approximately 70 h. To further assess the stability of the system, the steady-state performance was examined at a dilution rate of 0.04 h^−1^ over a period of 120 h, approaching five volume turnovers. Figure 7a illustrates good stability where the productivity and yield at a specific instant in the period remained within 4.3 and 3.8 % of the average productivity and yield, respectively. Minimal deviation in the productivity implies a consistent SA production rate and titre, while minimal deviation in the yield implies constant metabolic flux distributions indicating that the microbial population was indeed at steady state. This further suggests that the active biomass content in the fermenter was constant during this interval. However, further increasing the dilution rate to 0.05 h^−1^ led to a significant decrease in *C*_SA_ after which the fermentation was terminated. The first fermentation thus provided important insights into the response of *A. succinogenes* to the throughput rates of non-detoxified DDAP-H, and suggests that a gradual increase in *D* is vital in maintaining culture viability.

Based on the conclusions of the first DDAP-H fermentation, the strategy of the second DDAP-H fermentation was to operate at a low dilution rate for an extended period of time (approximately 100-h holding times), followed by dilution rate increases in increments of 0.01 h^−1^. Figure 6b shows that by operating at *D* = 0.01 h^−1^ after the initial batch phase for approximately 90 h, then increasing D incrementally after holding times of approximately 100 h, results in a steady increase in *C*_SA_ up to 36.6 g L^−1^, followed by a stable value of 35.3 g L^−1^ on average. Therefore, by gradually increasing *D*, the culture was able to acclimatise sufficiently for stable operation at practical dilution rates and unlike in the first DDAP-H run, the system did not destabilise at 0.05 h^−1^. Instead, the system showed good stability at an average *C*_SA_ of around 33.3 g L^−1^, and similar to the above analysis, the productivity and yield fluctuated minimally around the averages remaining within 2.1 and 2.9 %, respectively (Fig. 7b). Furthermore, the biofilm appeared stable throughout the fermentation as no major shedding or sloughing events occurred and the effluent did not contain large clumps of biomass. It is plausible that the thickness of biofilm and biofilm buildup on the fitting were limited by shear effects through sufficient agitation, in addition to product inhibition.

**Fig. 8:**
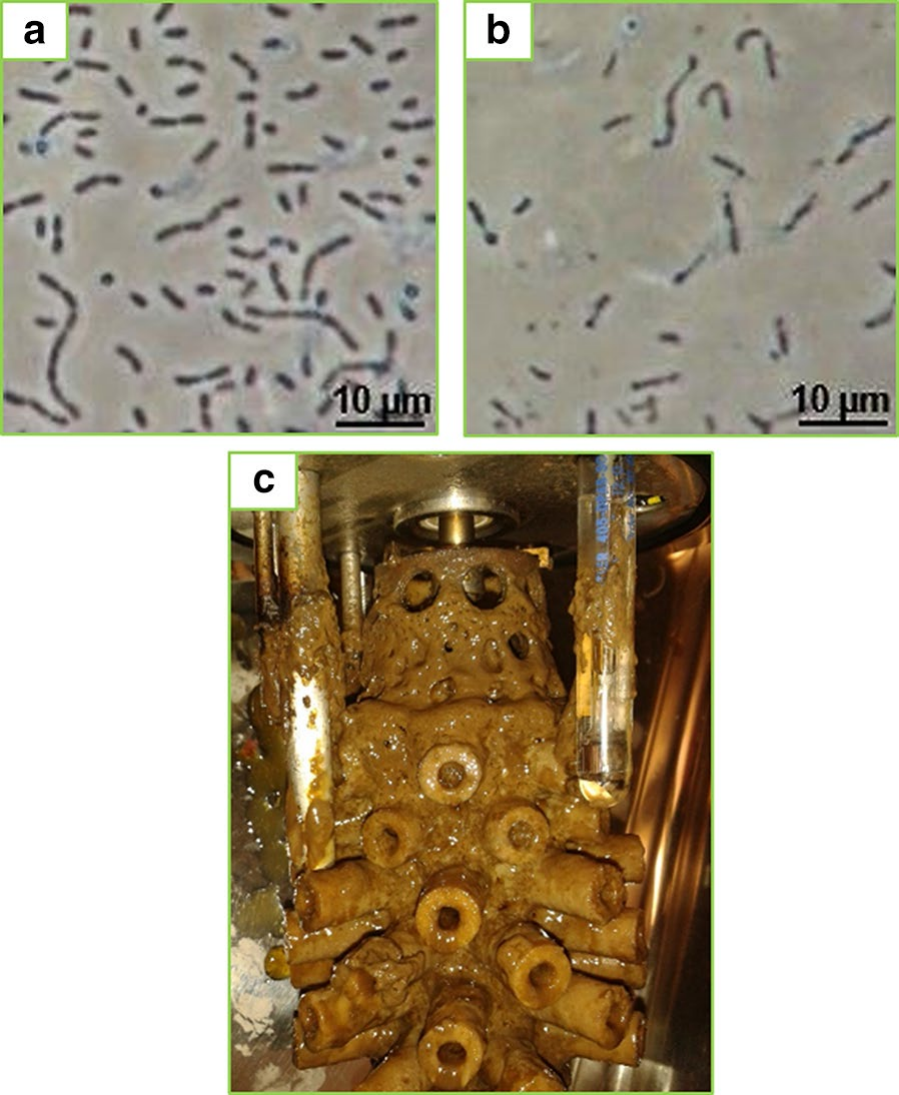
Microscope images and biofilm from the DDAP-H fermentations. Microscope images from the first DDAP-H fermentation: **a** during the batch start-up phase, and **b** after continuous operation at *D* = 0.02 h^−1^ for 64 h (approx. 167 h fermentation time); **c** biofilm attached to the agitator fitting and reactor internals as seen at the termination of the first DDAP-H fermentation

Interestingly, the cell morphology was found to differ between batch (Fig. 8a) and continuous modes (Fig. 8b) where cells exhibited a more elongated, irregular shape during continuous operation as compared to batch. The irregular shape could be indicative of stress caused by the high acid titres or merely from washed out fragments of biofilm. As a comparison to Figs. 1b and 8c show the agitator fitting after termination of the first DDAP-H run where biofilm attachment is clearly visible on the centre PP tube and the porous PP protruding arms. Overall, the second fermentation demonstrated that *A. succinogenes* is able to effectively convert non-detoxified DDAP-H into succinic acid, given a gradual increase in the dilution rate to allow the organism to adapt to the inhibitors in the hydrolysate, whilst showing good overall stability and sustainable steady-state conditions.

## Conclusions

Value-added chemicals produced in conjunction to biofuels are important in reducing the risks inherent in the overall economics of a lignocellulosic biorefinery. Production of such chemicals requires co-development with upstream and downstream processes including pretreatment and separation. It is therefore necessary to assess the performance of co-production fermentation processes using a process-relevant feed stream and with consideration of downstream requirements. In this work, a target value-added chemical—succinic acid—was produced continuously as the major end product by immobilised *A. succinogenes* at competitive productivities (1.77 g L^−1^ h^−1^), yields (0.78 g g^−1^) and titres (39.6 g L^−1^) on a non-detoxified, xylose-rich hydrolysate stream for the first time. The productivities attained in this study exceed those of similar studies whilst achieving similar yields and titres over prolonged periods of operation. High productivities were possible due to high cell densities achieved through immobilisation of cells as a biofilm on a novel agitator fitting, and through continuous operation of the fermenter. Ultimately, effective conversion of a process-relevant, lignocellulose-derived hydrolysate stream at high succinate production rates, and titres favourable for downstream separation processes, is demonstrated. Therefore, this work illustrates that co-production of value-added chemicals is feasible by microbial conversion of biorefinery streams, and provides a baseline for similar studies in the future.

## Methods

### Microorganism

Wild-type *Actinobacillus succinogenes* 130Z (ATCC 55618; DSM 22257) [24] was acquired from the American Type Culture Collection. Culture samples were stored at −80 °C in a cryopreservation solution (40 % glycerol solution mixed with an equal volume of cells). Inoculum was prepared by reviving a frozen culture in a 30 g L^−1^ tryptone soy broth (TSB) (Sigma-Aldrich, USA) solution supplemented with 1 % glucose and incubating for 16–24 h at 37 °C and 100 rpm in a sealed septum vial. In the case of hydrolysate fermentations, a 1:3 TSB-hydrolysate solution was used in inoculation preparation to allow the culture to acclimatise to the hydrolysate. Purity and viability of the inoculum were determined by microscopy and high-performance liquid chromatography (HPLC) where the presence of SA, AA and FA indicated viability and the absence of lactic acid (or other unexpected compounds) indicated purity.

### Hydrolysate preparation

Corn stover harvested in Emmetsburg, IA, USA underwent deacetylation, followed by acid impregnation and pilot-scale pretreatment, as described in [66]. Deacetylation was performed by mixing dry corn stover with a 0.4 % w/w sodium hydroxide solution and holding for 2 h at 80 °C. Following this, a dilute solution of sulphuric acid (0.8 % w/w) was added to drained solids from the deacetylation process for acid impregnation, after which the acid-impregnated solids were mixed at room temperature for 2 h followed by dewatering using a screw press. The deacetylated, acid-impregnated corn stover then underwent pilot-scale acid hydrolysis pretreatment in a horizontal pretreatment reactor (Metso Inc., USA) at 150–170 °C with residence times of 10–20 min. The resulting deacetylated corn stover hydrolysate (DDAP-H) was stored in drums at 5 °C. Prior to preparation of the fermentation medium, the xylose-rich liquid fraction of the DDAP-H was separated from the glucose-rich solids fraction by means of a mechanical press. The liquid fraction was utilised in this study with the average composition in the fermenter feed stream given in Table 2.

### Fermentation medium

The fermentation medium, a simplified version of the medium used by Bradfield and Nicol [15], consisted of three parts: (A) a growth and salts solution, (B) a phosphate buffer solution and, (C) a carbohydrate solution. Part A comprised (in g L^−1^): 0.2 MgCl_2_, 0.2 CaCl_2_·2H_2_O, 0.5 NaCl (Fisher Scientific, USA), 6.0 yeast extract powder (BD, USA), 10.0 clarified corn steep liquor and 0.5 mL L^−1^ antifoam SE-15. Part B consisted of 1.6 g L^−1^ KH_2_PO_4_ and 0.8 g L^−1^ K_2_HPO_4_. The carbohydrate solution consisted of either xylose at 60 g L^−1^ or de-acetylated corn stover hydrolysate at a total sugars concentration of 70 g L^−1^ (including xylose, glucose, arabinose and galactose). Note, all chemicals were obtained from Sigma-Aldrich (USA), unless indicated otherwise, and the concentration values are given for the overall (combined) solution volume.

Corn steep liquor was clarified by boiling a 200-g L^−1^ solution for 8 min at 121 °C in an autoclave. After approximately 24 h—once the bulk of the solids had settled out through gravity—the supernatant was removed and stored at 5 °C. The supernatant was used as the clarified corn steep liquor in Part A of the medium. The DDAP-H was passed through a 0.2 µm filter to remove any spores prior to preparing the solution.

### Fermentation

All fermentations were performed using a 1.6-L BioFlo 3000 bioreactor system (New Brunswick Scientific, USA). The working volume (overall reactor volume based on the vessel size) was controlled at 1.3 L by means of an overflow tube connected to an exit pump. A liquid-free headspace assisted with foam control. To increase the available surface area for cell attachment and biofilm growth, a novel agitator fitting was developed (Fig. 1). The fitting comprised a central porous polypropylene (PP) tube perforated with a multitude of threaded holes into which porous PP or silicone protruding arms were attached to provide additional surface area and sufficient agitation ability. The central tube was attached to the agitation shaft by means of stainless steel brackets. Silicone arms were used in the xylose fermentation, whereas porous PP arms were used in the DDAP-H fermentations since good adherence to the inner PP tube was demonstrated in the xylose fermentations, and it was expected that porous PP would provide greater surface area than solid silicone for attachment.

CO_2_ (General Air, USA) supply to the fermenter was controlled manually at a fixed rate of 0.10 vvm by means of a 65-mm aluminium rotameter (Cole-Parmer, USA), and fed through a submerged sparger located beneath the agitation shaft. All gas entering and exiting the fermenter, and venting from reservoirs, passed through Millex-FG 0.2 µm PTFE filters (Millipore, USA) to ensure sterility. Gas vented through the head of the fermenter was passed through a drainable foam trap to prevent blockage of the vent filter. Quantification of foam volume (foam overflow into the foam trap) contributed to overall dilution rate calculations in instances of extensive foaming. Temperature was controlled at 37 °C by means of a thermocouple, housed within a stainless steel cover submerged in the fermenter, coupled to a PID controller within the BioFlo system. pH was controlled at 6.80 using a gel-filled 405-DPAS probe (Mettler Toledo, Switzerland) coupled to a PID controller which regulated the dosing of an unsterilised 10 N NaOH solution (Fisher Scientific, USA). A 10 % v/v solution of antifoam SE-15 (Sigma-Aldrich, USA) was dosed as needed into the headspace during operation to suppress foaming.

The complete fermenter setup (reactor, tubing, and reservoirs) was autoclaved at 121 °C for 60–75 min (depending on the volume of feed used), with the three medium parts kept in separate bottles to prevent unwanted reactions (e.g. Maillard reactions and phosphate precipitation) during sterilisation. Once the system had cooled, the medium parts were mixed into a single bottle. In the case of hydrolysate fermentations, the reactor contained approximately 1 L of a TSB-hydrolysate mixture (3:1) during autoclaving to serve as a start-up growth medium and to ensure that the pH probe remained wet. Similar to the inoculum preparation, hydrolysate was included to facilitate adaptation of the culture to the hydrolysate thereby avoiding shocking the organism when the fermenter was switched to the DDAP-H stream. The fermentation was initialised by operating in batch mode for 16–24 h after inoculation and once the sugar concentrations were sufficiently low, the system was switched to continuous mode by feeding the fermentation medium at a low dilution rate to avoid cell washout. Since the TSB/hydrolysate mixture within the fermenter only features during the start-up phase of the process, it is unlikely that potentially unwanted reactions caused by autoclaving the mixture (e.g. Maillard reactions) will have an influence on the steady-state results under continuous conditions. Also, steady-state conditions occurred long after the start-up batch; therefore, the TSB/hydrolysate mixture would have been extensively diluted or completely washed out at this point. The first fermentation was performed using xylose (~60 g L^−1^) as the only carbohydrate substrate to establish a baseline for the DDAP-H fermentations and to test the ability of the agitator fittings to increase cell density. The DDAP-H fermentations were repeated in duplicate and steady states were obtained at dilution rates of 0.02, 0.03–0.04 h^−1^ with a combined fermentation time of approximately 1550 h. A single-steady state was achieved at 0.05 h^−1^ in the second DDAP-H fermentation.

### Analytical methods

High-performance liquid chromatography (HPLC) was used to analyse the composition of the fermentation medium and the fermenter outlet. Organic acids and fermentation inhibitors (HMF and furfural) were detected by means of an Agilent 1100 system (Agilent Technologies, USA) fitted with an refractive index detector (RID) and an Aminex HPX-87H ion-exchange column (Bio-Rad Laboratories, USA). The mobile phase was 0.01 N H_2_SO_4_ at a flow rate of 0.6 mL min^−1^. Column and RID temperatures were maintained at 85 and 55 °C, respectively. A sample injection volume of 6 µL was used. Carbohydrates (glucose, xylose, arabinose and galactose) were detected using the same system type and parameters as before except with a Phenomenex SP0810 column (Phenomenex, USA) and deionised water as the mobile phase. A YSI 7100 MBS (YSI Life Sciences, USA) was used for glucose and xylose detection at low concentrations due to reduced sensitivity of the respective HPLC system at low sugar concentrations.

Analysis of phenolic compounds from the feed and outlet dilutions was performed on an Agilent 1100 system equipped with a G1315B diode array detector (DAD) and an Ion Trap SL (Agilent Technologies, USA) mass spectrometer (MS) with in-line electrospray ionisation (ESI). Each sample was injected undiluted at a volume of 50 μL into the LC/MS system. Compounds were separated using an YMC C30 Carotenoid 0.3 μm, 4.6 × 150 mm column (YMC America, USA) at an oven temperature of 30 °C. The chromatographic eluents consisted of (A) water modified with 0.03 % formic acid, and (B) 9:1 acetonitrile and water also modified with 0.03 % formic acid. At a flow rate of 0.7 mL min^−1^, the eluent gradient was as follows: 0–3 min, 0 % B; 16 min, 7 % B; 21 min, 8.5 % B; 34 min, 10 % B; 46 min, 25 % B; 51–54 min, 30 % B; 61 min, 50 % B; and lastly 64–75 min, 100 % B before equilibrium. Deionized water (Barnstead Easy Pure^II^, USA), acetonitrile (HPLC grade, Fisher Scientific, USA), and formic acid with a purity of 98 % (Sigma-Aldrich, USA) were used as HPLC solvents and modifiers.

Flow from the HPLC–DAD was directly routed in series to the ESI–MS ion trap. The DAD was used to monitor chromatography at 210 nm for a direct comparison to MS data. Source and ion trap conditions were calibrated with Agilent ESI-T tuning mix (P/N:G2431A), while tuning parameters were optimised under negative-ion mode by direct infusion of standards for major contributing compounds. MS and MS/MS parameters are as follows: smart parameter setting with target mass set to 165 Da, compound stability 70 %, trap drive 50 %, capillary at 3500 V, fragmentation amplitude of 0.75 V with a 30–200 % ramped voltage implemented for 50 ms, and an isolation width of 2 m/z (He collision gas). The ESI nebulizer gas was set to 60 psi, with dry gas flow of 11 L min^−1^ held at 350 °C. MS scans and precursor isolation-fragmentation scans were performed across the range of 40–350 Da.

### Data analysis and collection

Online monitoring of the process parameters was performed by means of BioCommand software (New Brunswick Scientific, USA). The weighted time average of the NaOH flow rate was calculated in real time and used as an indication of steady state. Once the time-averaged NaOH flow rate remained within 5 % of the average over at least a 24-h period, and sugar and metabolite concentrations remained within 3 % over the same interval, the system was considered to be at pseudo steady state. Furthermore, samples were taken daily to assess the transient behaviour of the system. The time-averaged NaOH flow rate, together with the antifoam flow rate, was used in calculating a dilution factor to adjust the inlet concentration of the substrates and other compounds of relevance due to dilution by the additional flow.

The accuracy and completeness of the data were assessed by performing overall mass balances. Mass balances were performed by calculating the stoichiometric amount of carbohydrates required to produce the measured metabolite concentrations based on elemental balances, and comparing this amount to the actual (measured) amount of carbohydrates consumed. Since the carbohydrates all have the same C:H:O ratio, the carbohydrates were combined into a single amount in the calculation. The percentage closure of the mass balance is calculated as the ratio of the required amount of carbohydrates consumed to the measured amount of carbohydrates consumed. A value less than 100 implies that more carbon was consumed than accounted for by the metabolites and cell mass.

## Abbreviations

AA: Acetic acid
*C*_AA_: Acetic acid concentration (g L^−1^)
*C*_FA_: Formic acid concentration (g L^−1^)
*C*_SA_: Succinic acid concentration (g L^−1^)
CSL: Corn steep liquor
*D*: Dilution rate (h^−1^)
DAP: Dilute acid pretreatment
DAP-H: Dilute acid pretreated hydrolysate
DDAP-H: Deacetylated, dilute acid pretreated corn stover hydrolysate
FA: Formic acid
HMF: Hydroxymethylfurfural
OPPP: Oxidative pentose phosphate pathway
PP: Polypropylene
*q*_SA_: Succinic acid productivity (g L^−1^ h^−1^)
SA: Succinic acid
TSB: Tryptone soy broth
*Y*_AAFA_: Formic acid to acetic acid ratio (g g^−1^)
*Y*_AASA_: Succinic acid to acetic acid ratio (g g^−1^)
*Y*_sSA_: Yield of SA on sugars (g g^−1^)
